# Myalgic encephalomyelitis: International Consensus Criteria

**DOI:** 10.1111/j.1365-2796.2011.02428.x

**Published:** 2011-08-22

**Authors:** B M Carruthers, M I van de Sande, K L De Meirleir, N G Klimas, G Broderick, T Mitchell, D Staines, A C P Powles, N Speight, R Vallings, L Bateman, B Baumgarten-Austrheim, D S Bell, N Carlo-Stella, J Chia, A Darragh, D Jo, D Lewis, A R Light, S Marshall-Gradisbik, I Mena, J A Mikovits, K Miwa, M Murovska, M L Pall, S Stevens

**Affiliations:** 1 IndependentVancouver, BC, Canada; 2 IndependentCalgary, AB, Canada; 3 Department of Physiology and Medicine, Vrije University of Brussels, Himmunitas FoundationBrussels, Belgium; 4 Department of Medicine, University of Miami Miller School of Medicine and Miami Veterans Affairs Medical CenterMiami, FL, USA; 5 Department of Medicine, University of AlbertaEdmonton, AB, Canada; 6 Honorary Consultant for NHS at Peterborough/CambridgeLowestoft, Suffolk, UK; 7 Gold Coast Public Health UnitSouthport, Queensland; 8 Health Sciences and Medicine, Bond UniversityRobina, Queensland, Australia; 9 Faculty of Health Sciences, McMaster University and St. Joseph's Healthcare HamiltonHamilton, ON, Canada; 10 IndependentDurham, UK; 11 Howick Health and Medical CentreHowick, New Zealand; 12 Fatigue Consultation Clinic, Salt Lake Regional Medical CenterSalt Lake City, UT, USA; 13 Internal Medicine, Family Practice, University of UtahSalt Lake City, UT, USA; 14 ME/CFS Center, Oslo University Hospital HFNorway; 15 Department of Paediatrics, State University of New YorkBuffalo, NY, USA; 16 IndependentPavia, Italy; 17 Harbor-UCLA Medical Center, University of CaliforniaLos Angeles, CA; 18 EV Med ResearchLomita, CA, USA; 19 University of LimerickLimerick, Ireland; 20 Pain Clinic, Konyang University HospitalDaejeon, Korea; 21 Donvale Specialist Medical CentreDonvale, Victoria, Australia; 22 Departments of Anesthesiology, Neurobiology and Anatomy, University of UtahSalt Lake City, UT, USA; 23 Department of Medicina NuclearClinica Las Condes, Santiago, Chile; 24 Whittemore Peterson Institute, University of NevadaReno, NV, USA; 25 Miwa Naika ClinicToyama, Japan; 26 A. Kirchenstein Institute of Microbiology and Virology, Riga Stradins UniversityRiga, Latvia; 27 Department of Biochemistry & Basic Medical Sciences, Washington State UniversityPortland, OR; 28 Department of Sports Sciences, University of the PacificStockton, CA USA

**Keywords:** chronic fatigue syndrome, criteria, definition, diagnosis, myalgic encephalomyelitis

## Abstract

Carruthers BM, van de Sande MI, De Meirleir KL, Klimas NG, Broderick G, Mitchell T, Staines D, Powles ACP, Speight N, Vallings R, Bateman L, Baumgarten-Austrheim B, Bell DS, Carlo-Stella N, Chia J, Darragh A, Jo D, Lewis D, Light AR, Marshall-Gradisbik S, Mena I, Mikovits JA, Murovska M, Pall ML, Stevens S (Independent, Vancouver, BC, Canada; Independent, Calgary, AB, Canada; Department of Physiology and Medicine, Vrije University of Brussels, Himmunitas Foundation, Brussels, Belgium; Department of Medicine,University of Miami Miller School of Medicine and Miami Veterans Affairs Medical Center, Miami, FL, USA; Department of Medicine, University of Alberta, Edmonton, AB, Canada; Honorary Consultant for NHS at Peterborough/Cambridge, Lowestoft, Suffolk, UK; Gold Coast Public Health Unit, Southport, Queensland; Health Sciences and Medicine, Bond University, Robina, Queensland, Australia; Faculty of Health Sciences, McMaster University and St Joseph’s Healthcare Hamilton, Hamilton, ON, Canada; Independent, Durham, UK; Howick Health and Medical Centre, Howick, New Zealand; Fatigue Consultation Clinic, Salt Lake Regional Medical Center; Internal Medicine, Family Practice, University of Utah, Salt Lake City, UT, USA; ME/CFS Center, Oslo University Hospital HF, Norway; Department of Paediatrics, State University of New York, Buffalo, NY; Independent, Pavia, Italy; Harbor-UCLA Medical Center, University of California, Los Angeles, CA; EV Med Research, Lomita, CA, USA; University of Limerick, Limerick, Ireland; Pain Clinic, Konyang University Hospital, Daejeon, Korea; Donvale Specialist Medical Centre, Donvale, Victoria, Australia; Departments or Anesthesiology, Neurobiology and Anatomy, University of Utah, Salt Lake City, Utah, USA; Health Sciences and Medicine, Bond University, Robina, Queensland, Australia; Department of Medicina Nuclear, Clinica Las Condes, Santiago, Chile; Whittemore Peterson Institute, University of Nevada, Reno, NV, USA; Miwa Naika Clinic, Toyama, Japan; A. Kirchenstein Institute of Microbiology and Virology, Riga Stradins University, Riga, Latvia; Department of Biochemistry & Basic Medical Sciences, Washington State University, Portland, OR; Department of Sports Sciences, University of the Pacific, Stockton, CA USA). Myalgic encephalomyelitis: International Consensus Criteria (Review). *J Intern Med* 2011; **270**: 327–338.

The label ‘chronic fatigue syndrome’ (CFS) has persisted for many years because of the lack of knowledge of the aetiological agents and the disease process. In view of more recent research and clinical experience that strongly point to widespread inflammation and multisystemic neuropathology, it is more appropriate and correct to use the term ‘myalgic encephalomyelitis’ (ME) because it indicates an underlying pathophysiology. It is also consistent with the neurological classification of ME in the World Health Organization’s International Classification of Diseases (ICD G93.3). Consequently, an International Consensus Panel consisting of clinicians, researchers, teaching faculty and an independent patient advocate was formed with the purpose of developing criteria based on current knowledge. Thirteen countries and a wide range of specialties were represented. Collectively, members have approximately 400 years of both clinical and teaching experience, authored hundreds of peer-reviewed publications, diagnosed or treated approximately 50 000 patients with ME, and several members coauthored previous criteria. The expertise and experience of the panel members as well as PubMed and other medical sources were utilized in a progression of suggestions/drafts/reviews/revisions. The authors, free of any sponsoring organization, achieved 100% consensus through a Delphi-type process. The scope of this paper is limited to criteria of ME and their application. Accordingly, the criteria reflect the complex symptomatology. Operational notes enhance clarity and specificity by providing guidance in the expression and interpretation of symptoms. Clinical and research application guidelines promote optimal recognition of ME by primary physicians and other healthcare providers, improve the consistency of diagnoses in adult and paediatric patients internationally and facilitate clearer identification of patients for research studies.

## Introduction

Myalgic encephalomyelitis (ME), also referred to in the literature as chronic fatigue syndrome (CFS), is a complex disease involving profound dysregulation of the central nervous system (CNS) [1–3] and immune system [4–8], dysfunction of cellular energy metabolism and ion transport [9–11] and cardiovascular abnormalities [12–14]. The underlying pathophysiology produces measurable abnormalities in physical and cognitive function and provides a basis for understanding the symptomatology. Thus, the development of International Consensus Criteria that incorporate current knowledge should advance the understanding of ME by health practitioners and benefit both the physician and patient in the clinical setting as well as clinical researchers.

The problem with broadly inclusive criteria [15, 16] is that they do not select homogeneous sets of patients. The Centers for Disease Control prevalence estimates increased tenfold from 0.24% using the Fukuda criteria [17] to 2.54% using the Reeves empirical criteria [16]. Jason *et al.* [18] suggest that there are flaws in Reeves’ methodology because it is possible to meet the empirical criteria for ME without having any physical symptoms and it does not discriminate patients with ME/CFS from those with major depressive disorder. Patient sets that include people who do not have the disease lead to biased research findings, inappropriate treatments and waste scarce research funds [19].

Some symptoms of the Fukuda criteria overlap with depression, whereas the Canadian Consensus Criteria [20] differentiate patients with ME from those who are depressed and identify patients who are more physically debilitated and have greater physical and cognitive functional impairments [21].

## International Consensus Criteria

The Canadian Consensus Criteria were used as a starting point, but significant changes were made. The 6-month waiting period before diagnosis is no longer required. No other disease criteria require that diagnoses be withheld until after the patient has suffered with the affliction for 6 months. Notwithstanding periods of clinical investigation will vary and may be prolonged, diagnosis should be made when the clinician is satisfied that the patient has ME rather than having the diagnosis restricted by a specified time factor. Early diagnoses may elicit new insights into the early stages of pathogenesis; prompt treatment may lessen the severity and impact.

Using ‘fatigue’ as a name of a disease gives it exclusive emphasis and has been the most confusing and misused criterion. No other fatiguing disease has ‘chronic fatigue’ attached to its name – e.g. cancer/chronic fatigue, multiple sclerosis/chronic fatigue – except ME/CFS. Fatigue in other conditions is usually proportional to effort or duration with a quick recovery and will recur to the same extent with the same effort or duration that same or next day. The pathological low threshold of fatigability of ME described in the following criteria often occurs with minimal physical or mental exertion and with reduced ability to undertake the same activity within the same or several days.

The International Consensus Criteria (Table 1) identify the unique and distinctive characteristic patterns of symptom clusters of ME. The broad spectrum of symptoms alerts medical practitioners to areas of pathology and may identify critical symptoms more accurately [18–20]. Operational notes following each criterion provide guidance in symptom expression and contextual interpretation. This will assist the primary clinician in identifying and treating patients with ME in the primary care setting.

**Table 1:** Myalgic encephalomyelitis: international consensus criteria

Adult and paediatric•clinical and research
*Myalgic encephalomyelitis is an acquired neurological disease with complex global dysfunctions. Pathological dysregulation of the nervous, immune and endocrine systems, with impaired cellular energy metabolism and ion transport are prominent features. Although signs and symptoms are dynamically interactive and causally connected, the criteria are grouped by regions of pathophysiology to provide general focus.*
A patient will meet the criteria for postexertional neuroimmune exhaustion (A), at least one symptom from three neurological impairment categories (B), at least one symptom from three immune/gastro-intestinal/genitourinary impairment categories (C), and at least one symptom from energy metabolism/transport impairments (D).
**A. Postexertional neuroimmune exhaustion (PENE pen’-e): Compulsory**
This cardinal feature is a pathological inability to produce sufficient energy on demand with prominent symptoms primarily in the neuroimmune regions. Characteristics are as follows:
**1. Marked, rapid physical and/or cognitive fatigability in response to exertion,** which may be minimal such as activities of daily living or simple mental tasks, can be debilitating and cause a relapse.
**2. Postexertional symptom exacerbation:** *e.g.acute flu-like symptoms, pain and worsening of other symptoms.*
**3.** **Postexertional exhaustion** may occur immediately after activity or be delayed by hours or days.
**4. Recovery period is prolonged,** usually taking 24 h or longer. A relapse can last days, weeks or longer.
**5. Low threshold of physical and mental fatigability (lack of stamina) results in a substantial reduction in pre-illness activity level.**
**Operational notes:** *For a diagnosis of ME, symptom severity must result in a significant reduction of a patient’s premorbid activity level. **Mild** (an approximate 50% reduction in pre-illness activity level), **moderate** (mostly housebound), **severe** (mostly bedridden) or **very severe** (totally bedridden and need help with basic functions). There may be marked fluctuation of symptom severity and hierarchy from day to day or hour to hour. Consider activity, context and interactive effects. **Recovery time**: e.g. Regardless of a patient’s recovery time from reading for ½ hour, it will take much longer to recover from grocery shopping for ½ hour and even longer if repeated the next day – if able. Those who rest before an activity or have adjusted their activity level to their limited energy may have shorter recovery periods than those who do not pace their activities adequately. **Impact**: e.g. An outstanding athlete could have a 50% reduction in his/her pre-illness activity level and is still more active than a sedentary person.*
**B. Neurological impairments**
**At least one symptom from three of the following four symptom categories**
**1. Neurocognitive impairments**
**a. Difficulty processing information:** slowed thought, impaired concentration *e.g. confusion, disorientation, cognitive overload, difficulty with making decisions*, *slowed speech, acquired or exertional dyslexia*
**b. Short-term memory loss:** *e.g*. *difficulty remembering what one wanted to say, what one was saying, retrieving words, recalling information*, *poor working memory*
**2. Pain**
**a. Headaches:** *e.g. chronic, generalized headaches often involve aching of the eyes, behind the eyes or back of the head that may be associated with cervical muscle tension; migraine; tension headaches*
**b. Significant pain** can be experienced in muscles, muscle-tendon junctions, joints, abdomen or chest. It is noninflammatory in nature and often migrates. *e.g. generalized hyperalgesia*, *widespread pain (may meet fibromyalgia criteria), myofascial or radiating pain*
**3. Sleep disturbance**
**a. Disturbed sleep patterns:** *e.g. insomnia, prolonged sleep including naps, sleeping most of the day and being awake most of the night, frequent awakenings, awaking much earlier than before illness onset, vivid dreams/nightmares*
**b. Unrefreshed sleep:** *e.g. awaken feeling exhausted regardless of duration of sleep, day-time sleepiness*
**4. Neurosensory, perceptual and motor disturbances**
**a. Neurosensory and perceptual:** *e.g*. *inability to focus vision, sensitivity to light, noise, vibration, odour, taste and touch; impaired depth perception*
**b. Motor:** *e.g. muscle weakness, twitching, poor coordination, feeling unsteady on feet, ataxia*
***Notes: Neurocognitive impairments**, reported or observed, become more pronounced with fatigue. **Overload phenomena** may be evident when two tasks are performed simultaneously. **Abnormal accommodation responses** of the pupils are common. **Sleep disturbances** are typically expressed by prolonged sleep, sometimes extreme, in the acute phase and often evolve into marked sleep reversal in the chronic stage. **Motor disturbances** may not be evident in mild or moderate cases but abnormal tandem gait and positive Romberg test may be observed in severe cases*.
**C. Immune, gastro-intestinal and genitourinary Impairments**
**At least one symptom from three of the following five symptom categories**
**1. Flu-like symptoms may be recurrent or chronic and typically activate or worsen with exertion.** *e.g. sore throat, sinusitis, cervical and/or axillary lymph nodes may enlarge or be tender on palpitation*
**2. Susceptibility to viral infections with prolonged recovery periods**
**3. Gastro-intestinal tract:** *e.g. nausea, abdominal pain, bloating, irritable bowel syndrome*
**4. Genitourinary:** e.g. *urinary urgency or frequency, nocturia*
**5. Sensitivities *to food, medications, odours or chemicals***
***Notes:*** *Sore throat, tender lymph nodes, and flu-like symptoms obviously are not specific to ME but their activation in reaction to exertion is abnormal. The throat may feel sore, dry and scratchy. Faucial injection and crimson crescents may be seen in the tonsillar fossae, which are an indication of immune activation.*
**D. Energy production/transportation impairments: At least one symptom**
**1. Cardiovascular:** *e.g. inability to tolerate an upright position - orthostatic intolerance, neurally mediated hypotension, postural orthostatic tachycardia syndrome*, *palpitations with or without cardiac arrhythmias, light-headedness/dizziness*
**2. Respiratory:** *e.g. air hunger, laboured breathing, fatigue of chest wall muscles*
**3. Loss of thermostatic stability:** *e.g. subnormal body temperature, marked diurnal fluctuations; sweating episodes, recurrent feelings of feverishness with or without low grade fever, cold extremities*
**4. Intolerance of extremes of temperature**
***Notes:*** *Orthostatic intolerance may be delayed by several minutes. Patients who have orthostatic intolerance may exhibit mottling of extremities, extreme pallor or Raynaud’s Phenomenon. In the chronic phase, moons of finger nails may recede.*
**Paediatric considerations**
*Symptoms may progress more slowly in children than in teenagers or adults. In addition to postexertional neuroimmune exhaustion, the most prominent symptoms tend to be neurological: headaches, cognitive impairments, and sleep disturbances.*
**1. Headaches:** Severe or chronic headaches are often debilitating. Migraine may be accompanied by a rapid drop in temperature, shaking, vomiting, diarrhoea and severe weakness.
**2. Neurocognitive impairments:** Difficulty focusing eyes and reading are common. Children may become dyslexic, which may only be evident when fatigued. Slow processing of information makes it difficult to follow auditory instructions or take notes. All cognitive impairments worsen with physical or mental exertion. Young people will not be able to maintain a full school programme.
**3. Pain may seem erratic and migrate quickly.** Joint hypermobility is common.
***Notes:*** *Fluctuation and severity hierarchy of numerous prominent symptoms tend to vary more rapidly and dramatically than in adults.*
**Classification**
**——— Myalgic encephalomyelitis**
**——— Atypical myalgic encephalomyelitis:** meets criteria for postexertional neuroimmune exhaustion but has a limit of two less than required of the remaining criterial symptoms. Pain or sleep disturbance may be absent in rare cases.
**Exclusions:** *As in all diagnoses, exclusion of alternate explanatory diagnoses is achieved by the patient’s history, physical examination, and laboratory/biomarker testing as indicated. It is possible to have more than one disease but it is important that each one is identified and treated.***Primary psychiatric disorders, somatoform disorder and substance abuse are excluded. Paediatric:**‘*primary’ school phobia.*
**Comorbid entities:** Fibromyalgia, myofascial pain syndrome, temporomandibular joint syndrome, irritable bowel syndrome, interstitial cystitis, Raynaud’s phenomenon, prolapsed mitral valve, migraines, allergies, multiple chemical sensitivities, Hashimoto’s thyroiditis, Sicca syndrome, reactive depression. *Migraine and irritable bowel syndrome may precede ME but then become associated with it. Fibromyalgia overlaps.*

## Criteria are supported by research

Criterial symptoms are supported by a study of more than 2500 patients that determined which symptoms had the greatest efficacy to identify patients with ME [22]. Investigations into gene expression [23–27] and structure further support the criteria at a molecular level including anomalies of increased oxidative stress [4, 28], altered immune and adrenergic signalling [29, 30] and altered oestrogen receptor expression [31]. In addition, evidence supporting a genetic predisposition to ME points to modifications in serotonin transporter genes [32, 33], the glucocorticoid receptor gene [34], as well as HLA class II involvement [35]. The potential combinatorial effects of these modifications have received limited attention [33, 36]. Some early broad-based studies show a lack of objective findings such as no association with HLA genotype [37]. A study of patients from a twin registry suggested that environmental factors may outweigh any genetic predisposition in broader patient populations [38].

Underlying problems of inconsistent findings in research studies have been identified [39, 40] and include a need for studies to be based on larger sample sizes with a more clearly defined phenotype, in particular one that recognizes the likely existence of significant subgroups within the patient population. In a study of the Reeves empirical criteria [16], Jason *et al.* [18] reported that 38% of patients diagnosed with major depressive disorder were misclassified as having CFS and only 10% of patients identified as having CFS actually had ME. Accordingly, the primary goal of this consensus report is to establish a more selective set of clinical criteria that would identify patients who have neuroimmune exhaustion with a pathological low threshold of fatigability and symptom flare in response to exertion. This will enable patients to be diagnosed and enrolled in research studies internationally under a case definition that is acceptable to physicians and researchers around the world.

### Postexertional neuroimmune exhaustion (PENE pen′-e)

‘Malaise’– a vague feeling of discomfort or fatigue [41] – is an inaccurate and inadequate word for the pathological low-threshold fatigability and postexertional symptom flare. Pain and fatigue are crucial bioalarm signals that instruct patients to modify what they are doing in order to protect the body and prevent further damage. Postexertional neuroimmune exhaustion is part of the body’s global protection response and is associated with dysfunction in the regulatory balance within and between the nervous, immune and endocrine systems, and cellular metabolism and ion transport [42–46]. The normal activity/rest cycle, which involves performing an activity, becoming fatigued and taking a rest whereby energy is restored, becomes dysfunctional.

Numerous papers document abnormal biological responses to exertion, such as loss of the invigorating effects of exercise [20], decreased pain threshold [47–49], decreased cerebral oxygen and blood volume/flow [50–53], decreased maximum heart rate [54], impaired oxygen delivery to muscles [55], elevated levels of nitric oxide metabolites [56] and worsening of other symptoms [57]. Patients reach the anaerobic threshold and maximal exercise at a much lower oxygen consumption level [58]. Reported prolonged effects of exertion include elevated sensory signalling to the brain [59] that is interpreted as pain and fatigue [29], elevated cytokine activity [60], delay in symptom activation [61] and a recovery period of at least 48 h [57]. When an exercise test was given on two consecutive days, some patients experienced up to a 50% drop in their ability to produce energy on the second evaluation [62]. Both submaximal and self-paced physiologically limited exercise resulted in postexertional malaise [48].

### Neurological impairments

Some viruses and bacteria can infect immune and neural cells and cause chronic inflammation. Structural and functional pathological abnormalities [3] within the brain and spinal cord suggest dysregulation of the CNS control system and communication network [62], which play crucial roles in cognitive impairment and neurological symptoms [20]. Neuroinflammation of the dorsal root ganglia, gatekeepers of peripheral sensory information travelling to the brain, has been observed in spinal autopsies (Chaudhuri A. Royal Society of Medicine Meeting 2009). Identified cerebrospinal fluid proteomes distinguish patients from healthy controls and post-treatment Lyme disease [63]. Neuroimaging studies report irreversible punctuate lesions [64], an approximate 10% reduction in grey matter volume [65, 66], hypoperfusion [50, 67–71] and brain stem hypometabolism [1]. Elevated levels of lateral ventricular lactate are consistent with decreased cortical blood flow, mitochondrial dysfunction and oxidative stress [72]. Research suggests that dysregulation of the CNS and autonomic nervous system alters the processing of pain and sensory input [29, 47, 73, 74]. Patients’ perception that simple mental tasks require substantial effort is supported by brain scan studies that indicate greater source activity and more regions of the brain are utilized when processing auditory and spatial cognitive information [75–77]. Poor attentional capacity and working memory are prominent disabling symptoms [20, 75, 78].

### Immune impairments

Most patients have an acute infectious onset with flu-like and/or respiratory symptoms. A wide range of infectious agents have been reported in the subsets of patients, including xenotropic murine leukaemia virus-related virus (XMRV) [79] and other murine leukaemia virus (MLV)-related viruses [80], enterovirus [81–83], Epstein–Barr virus [84], human herpes virus 6 and 7 [85–87], Chlamydia [88], cytomegalovirus [89], parvovirus B19 [90] and Coxiella burnetti [84]. Chronic enterovirus infection of the stomach and altered levels of D Lactic acid-producing bacteria in the gastrointestinal tract have been investigated [82, 91]. Possibly, the initial infection damages part of the CNS and immune system causing profound deregulation and abnormal responses to infections [4]. Publications describe decreased natural killer cell signalling and function, abnormal growth factor profiles, decreased neutrophil respiratory bursts and Th1, with a shift towards a Th2 profile [4–8, 92, 93]. Chronic immune activation [27], increases in inflammatory cytokines, pro-inflammatory alleles [4–8, 94–96], chemokines and T lymphocytes and dysregulation of the antiviral ribonuclease L (RNase L) pathway [62, 97–100] may play a role in causing flu-like symptoms, which aberrantly flare in response to exertion [5, 92].

### Energy production/transport impairments

The consistent clinical picture of profound energy impairment suggests dysregulation of the mitochondria and cellular energy metabolism and ion transport and channelopathy [9–11, 100, 101]. A biochemical positive feedback cycle called the ‘NO/ONOO- cycle’ may play a role in maintaining the chronic nature of ME, the presence of oxidative stress [102–104], inflammatory cytokine elevation [94–96] and mitochondrial dysfunction [105–108] and result in reduced blood flow and vasculopathy [106, 107].

Findings of ‘small heart’ with small left ventricular chamber and poor cardiac performance in patient subsets [109, 110] support previous reports of cardiac and left ventricular dysfunction [13, 111, 112], which predispose to orthostatic intolerance [14, 113]. Low blood pressure and exaggerated diurnal variation may be due to abnormal blood pressure regulation [114]. Altered control and reduced cortisol production during and following exercise may be involved. Orthostatic intolerance is associated with functional impairment and symptom severity [115]. Measurable vascular abnormalities suggest that the brain is not receiving sufficient circulating blood volume in an upright position [12, 113], which is intensified when standing in one place such as a grocery store check-out line. Significant reduction in heart rate variability during sleep is associated with poor sleep quality and suggests a pervasive state of nocturnal sympathetic hypervigilance [116].

## Application of criteria

Diagnostic criteria serve two necessary but divergent functions – the first is diagnosing individuals in a clinical setting and the second is identifying patient sets for research studies.

### Clinical application

#### General considerations

1. *Determine whether symptom cluster patterns are congruent* with those expected from dysfunction of an underlying causal system.
2. *Symptoms interact dynamically* within a stable cluster because they share the same deep causal roots. Patients’ contextual observations are essential in determining the expression of interaction of symptom patterns and severity of their impact.
3. *Symptom severity impact* must result in a 50% or greater reduction in a patient’s premorbid activity level for a diagnosis of ME. Mild: approximately 50% reduction in activity, moderate: mostly housebound, severe: mostly bedbound and very severe: bedbound and dependent on help for physical functions.
4. *Symptom severity hierarchy* should be determined periodically to help orient and monitor treatment.
5. *Criterial subgroups:* Postexertional neuroimmune exhaustion is the hallmark feature. It may be helpful to subgroup according to which of the other diagnostic criterial patterns best represent a patient’s cluster of most severe symptoms: neurological, immune, energy metabolism/transport or eclectic (symptoms widely distributed amongst subgroups).
6. *Separate primary symptoms from secondary symptoms and aggravators.* Distinguish primary symptom complexes formed by a disease process from secondary effects of coping with the disease, such as anxiety about finances. Determine the effects and burden of aggravators and stress enhancers such as fast paced environments and exposure to toxins.
7. *Determine total illness burden by assessing symptom severity, interaction and overall impact.* Consider all aspects of the patient’s life – physical, occupational, educational, social and personal activities of daily living. Patients who prioritize their activities may be able to do one important activity by eliminating or severely reducing activities in other aspects of their life.
8. *The International Symptom Scale* should not be part of the initial clinical interview because it may disturb the weighting and significance of results obtained for an individual patient. When used periodically, it can help position the patient within the group, orient the treatment programme and monitor its effectiveness.

#### Paediatric considerations

1. If possible, interview a young person with both parents because each may remember different symptoms or interactive events that may help determine onset and when the illness began to interfere with daily function.
2. Children cannot be expected to judge pre-illness function with current function. Assess impact by comparing hobbies, educational, social and sport activities the child participated in before illness with present activity level.
3. Children may appear irritable when they are asked to do something when they feel exhausted. On the other hand, they are often able to accommodate fatigue by resting, which may be inappropriately interpreted as being lazy.
4. *School Phobia:* Young patients spend most of their out-of-school hours resting, whereas children with school phobia will be socializing and participating in activities. However, it is possible that school phobia may become a secondary symptom because of bullying or academic difficulties owing to having ME.
5. *Natural Course:* Children can be very severely afflicted but those whose symptoms are of mild to moderate severity generally are more likely to have them go into remission than adults. Prognosis cannot be predicted with certainty.

### Research application

A clinical diagnosis must be confirmed before a patient can provide useful general knowledge about the disease. The data obtained from patients allow controlled and meaningful observations and suggest hypotheses to be tested and confirmed or refuted.

#### General considerations

1. *Patients should meet the full criteria* for epidemiological studies. If specific subgroups or atypical ME are included in a research study, that should be clearly indicated.
2. *Specificity:* Because critical symptoms are compulsory, it ensures proper selection of patients. Key operational guidelines enhance clarity and specificity. Ranking the hierarchy of the most troublesome symptoms may be helpful in some studies.
3. *Reliability:* Symptoms must not be viewed as a nominal checklist. The International Consensus Criteria focus on symptom patterns, which increase reliability. The International Symptom Scale ensures consistency in the way questions are asked and further increases the reliability of data collected in different locations. Patients should complete the International Symptom Scale prior to entering a research study.

#### Optional considerations

Classifying patients by subgroups to enable the comparison of patients within the diagnosis of ME may be helpful in some studies.

1. *Onset:* acute infectious or gradual.
2. *Onset severity* may be a good predictor of severity in the chronic phase.
3. *Symptom severity:* mild, moderate, severe, very severe.
4. *Criterial subgroups:* neurological, immune, energy metabolism/transport or eclectic.

(See clinical application for symptom severity and criterial subgroups.)

## Conclusions

The International Consensus Criteria provide a framework for the diagnosis of ME that is consistent with the patterns of pathophysiological dysfunction emerging from published research findings and clinical experience. Symptom patterns interact dynamically because they are causally connected. This has been formally addressed by some investigators who have used well-established multivariate statistical techniques, such as common factor or principal component analyses to identify symptom constructs [117, 118]. Others have extended the use of such methods to guide the analysis of gene expression profiles [28] and to delineate patient subgroups [119]. Consistent with this approach, the panel is developing an International Consensus Symptom Scale (ICSS) that will build on these underlying interactions. However, a necessary first step in establishing a quantitative score for any diagnostic instrument is the specification of measurable factors that are most relevant to the illness. Establishing such criteria was the primary objective of this work, and we believe the International Consensus Criteria will help clarify the unique signature of ME.

It is important to note that the current emphasis must primarily remain a clinical assessment, with selection of research subjects coming later. For this reason, the panel is developing Physicians’ Guidelines, which will include diagnostic protocol based on the International Consensus Criteria and treatment guidelines that reflect current knowledge. Individuals meeting the International Consensus Criteria have myalgic encephalomyelitis and should be removed from the Reeves empirical criteria and the National Institute for Clinical Excellence (NICE) criteria for chronic fatigue syndrome. These guidelines are designed specifically for use by the primary care physician in the hope of improving rapid diagnosis and treatment by first-line medical care providers. This may result in the development of an additional short-form version that would build on the relationships linking symptoms to formulate an abbreviated screening protocol. For the first time, clinical, paediatric and research applications are provided, which will advance the understanding of myalgic encephalomyelitis and enhance the consistency of diagnoses internationally. The compulsory critical criteria allow comparable data to be collected in various locations and may assist in developing consistent biomarkers and further insights into the mechanism and aetiology of myalgic encephalomyelitis.

## Funding

This Consensus paper is free of sponsorship. All authors contributed their time and expertise on a volunteer basis and no one received any payments or honorariums.

## Final approval and consensus

There was 100% consensus by the authors on the final consensus paper. BM Carruthers, MI van de Sande, KL De Meirleir, NG Klimas, G Broderick, T Mitchell, D Staines, ACP Powles, N Speight, R Vallings, L Bateman, B Baumgarten-Austrheim, DS Bell, N Carlo-Stella, J Chia, A Darragh, D Jo, D Lewis, AR Light, S Marshall-Gradisbik, I Mena, JA Mikovits, K Miwa, M Murovska, ML Pall, S Stevens.

## Consensus coordinator

M van de Sande.
